# Physical occupational exposures and health expectancies in a French occupational cohort

**DOI:** 10.1136/oemed-2016-103804

**Published:** 2016-09-21

**Authors:** Loretta G Platts, Jenny Head, Sari Stenholm, Holendro Singh Chungkham, Marcel Goldberg, Marie Zins

**Affiliations:** 1 Stress Research Institute, Stockholm University, Stockholm, Sweden; 2 Department of Epidemiology and Public Health, University College London, London, UK; 3 Department of Public Health, University of Turku and Turku University Hospital, Turku, Finland; 4 Indian Statistical Institute, North-East Centre, Tezpur, India; 5 Population-based Epidemiologic Cohorts Unit, UMS 011, Inserm, Villejuif, France; 6 Paris Descartes University, Paris, France; 7 UMR-S 1168 VIMA, Inserm, Villejuif, France

**Keywords:** chronic illness, self-rated health, work hazards

## Abstract

**Objectives:**

To examine the relationships of strenuous and hazardous working conditions and rotating shifts that involve night working with life expectancy in good perceived health and life expectancy without chronic disease.

**Methods:**

The sample contained male gas and electricity workers from the French GAZEL cohort (n=13 393). Six measures of physical working conditions were examined: Self-reports from 1989 and 1990 of ergonomic strain, physical danger, rotating shifts that involve night working and perceived physical strain; company records of workplace injuries and a job-exposure matrix of chemical exposures. Partial healthy life expectancies (age 50–75) relating to (1) self-rated health and (2) chronic health conditions, obtained from annual questionnaires (1989–2014) and company records, were estimated using multistate life tables. The analyses were adjusted for social class and occupational grade.

**Results:**

Participants with physically strenuous jobs and who had experienced industrial injuries had shorter partial life expectancy. More physically demanding and dangerous work was associated with fewer years of life spent in good self-rated health and without chronic conditions, with the exception of shift work including nights, where the gradient was reversed.

**Conclusions:**

Strenuous and hazardous work may contribute to lost years of good health in later life, which has implications for individuals' quality of life as well as healthcare use and labour market participation.

**What this paper adds**

- Physical working conditions cause a variety of illnesses and are associated with disability in later life. However, whether physical working conditions generate inequalities in health expectancies at older ages is not known.
- Using prospective longitudinal data, this study explores differences in life expectancy in good perceived health and life expectancy without chronic disease in relation to physical working conditions.
- More physically demanding and dangerous work was associated with more years lived in poor health and with chronic disease between the ages of 50 and 75 years.
- Strenuous and hazardous work may contribute to the burden of illness in later life.

## Introduction

While life expectancy has increased in recent decades,^1^ inequalities in longevity and health have endured. In particular, more advantaged people tend to have longer lives and longer healthy lives, differences which persist into old age.^2^ The lengthy and incremental development of chronic disease means that the major determinants of health and longevity in later life must most likely be sought earlier on in life. Capturing long-term, cumulative exposures is essential for understanding factors affecting morbidity and mortality in old age, particularly where they might be modifiable. One avoidable risk factor is exposure to poor physical working conditions, such as ergonomic strain, industrial injuries, exposure to dangerous substances and night shift work.

Previous work has convincingly demonstrated the role of physical working conditions in mortality and morbidity,^3–5^ but, to the best of our knowledge, little is known about the impact of physical working conditions on health expectancies. Therefore, this study examines the relationship between physical occupational exposures and health expectancies. A number of measures have been used to define the end of healthy life; therefore, this study uses two measures of health expectancy: (1) in good self-rated health and (2) without self-reported doctor-diagnosed chronic illnesses. Using prospective data from an occupational cohort, a longitudinal method based on the multistate life table approach is used to estimate health expectancies.^6^

This paper contributes to our understanding of the impact of physical working conditions on health expectancies. It examines whether those exposed to strenuous and hazardous working conditions as well as rotating shifts that involve night working experience a double disadvantage of shorter life expectancy and more years spent in poor health and with chronic disease.

## Methods

### Study population

The data come from the GAZEL cohort of employees of Électricité de France-Gaz de France (EDF-GDF), the French national utility company for the years 1989–2014. In 1989, male employees aged 40–50 years and female employees aged 35–50 years were invited to take part: 20 625 (or 44.7% of those eligible) did so.^7^ ^8^ Since its inception, participants have filled in annual postal self-completion questionnaires, covering a broad range of demographic, social and health topics, and the company has provided its personnel, medical and work exposure records. Information from company files, including for deaths, is near complete: The most recent published study of attrition from the GAZEL cohort found that after nearly 25 years only 2.6% of the initial participants had dropped out of the study, and around three-quarters of the participants return the annual questionnaires each year.^9^ The GAZEL cohort contains many participants who held manual occupations and have been exposed to strenuous and hazardous working conditions.

We included only male cohort members in the sample (n=15 011 men). A further 25 participants were excluded because they lacked company records, and 174 participants had died by or at the age of 50 years. There were some missing data, as a result of non-response and survey attrition: 133 people lacked information on at least 1 of the physical occupational exposures and a further 1306 participants had missing data for health or chronic conditions, leaving a final sample of 13 393 men.

The first year of the GAZEL survey was 1989. However, most participants were aged under 50 years in 1989, so the baseline year for a participant is the first year in which he is at least 50 years old and provides information on his health.

### Variables

#### Outcome variables

Two indicators of health were used to estimate health expectancies: self-rated health and self-reports of having a doctor-diagnosed chronic condition.

##### Self-rated health

At each of the annual questionnaire waves 1989–2014, participants responded to the question ‘How do you rate your general health status?’ by selecting an option on an eight-point scale with polar labels indicating very bad (1) or very good (8) health. The response options 5–8 were coded to indicate good health.^10^

##### Chronic conditions

The presence of the following chronic health conditions was ascertained in each of the annual questionnaires (1989–2014) with the question ‘has a doctor ever told you that you have…?’: (1) heart disease (heart attack, coronary heart disease, angina, congestive heart failure or other heart problems), (2) stroke (stroke or transient ischaemic attack), (3) chronic lung disease (chronic bronchitis, emphysema or asthma), (4) cancer (cancer or a malignant tumour of any kind except skin cancer), (5) diabetes (diabetes or high blood sugar) and (6) musculoskeletal disorders (arthritis or rheumatism). Individuals were defined as having a chronic health condition once they had ever reported one or more of these conditions. Individuals were also classified as having had a chronic health condition once they had been recorded as having a cancer in the company's medical register. This register, which has been validated for accuracy and completeness, records cancers occurring in actively employed, non-retired employees (1989–2014).^11^ The presence of chronic conditions at baseline (first observation included in the analysis) included any chronic conditions reported or recorded before the age of 50 from available information on respondents.

Mortality was ascertained from company records used for payment of pensions. Follow-up was censored at the end of 2014.

#### Physical occupational exposures

##### Ergonomic strain

In the 1989 and 1990 questionnaires, participants were asked whether their current work included any of five types of activities: (1) spending a long time on their feet, (2) spending a long time in another tiring posture, (3) long, frequent or rapid journeys in a vehicle, (4) carrying or moving heavy loads and (5) being subjected to shaking or vibrations. Affirmative responses to each item were summed into a 0–5 score of total ergonomic strain for each year.^12^ To minimise the influence of temporary activities and thereby reduce measurement error, the scores for 1989 and 1990 were averaged to produce a 1989/1990 score of ergonomic strain. If scores from both years were not available, the score from the year provided was used. Since it was not possible to know a priori where to set boundaries between categories, a three-category measure was created containing three roughly equally sized groups: no exposure, exposed at or below the median level of those exposed and exposed at more than the median level. The median level of exposure for those participants who reported ergonomic strain was 1, which corresponds to reporting a single exposure in 1989 and 1990, reporting no exposures in 1 year and two exposures in the other, or reporting a single exposure in 1 year and providing no report in the other.

##### Perceived physical strain

In the 1989 and 1990 questionnaires, participants were asked: ‘Do you find that your work is physically strenuous?’ and were provided with eight response options ranging from 1 (not at all strenuous) to 8 (very strenuous). This measure has been found to be a reliable and valid proxy measure for physical load at work, correlating with a more detailed scale of current and past postural constraints across a wide range of domains.^13^ The scores for 1989 and 1990 were averaged, and the measure of perceived physical strain was dichotomised at the median value of 3.5 for male participants.

##### Physical danger

In the 1989 and 1990 questionnaires, participants indicated whether they thought they were exposed to any of the following seven physical risks in the course of their work: (1) breathing in gas, (2) serious falls, (3) minor falls, (4) being injured by a machine, (5) heat burns, (6) chemical burns and (7) having a road accident. Affirmative responses to each item were summed to produce total scores of physical hazards in 1989 and 1990. In order to reduce the influence of short-term risks, as well as fluctuations in risk assessments, if scores for 1989 and 1990 were available, they were averaged to produce a single 0–7 score indicating exposure to physical hazards for the period 1989/1990. Otherwise, values from the year for which data were available were used. A three-category measure was created: no exposure, exposed at or below the median level (1.5 exposures) of those exposed and exposed at more than the median level.

##### Rotating shifts involving night shift work

Participants were asked in the 1989 and 1990 questionnaires whether their job involved working 8-hour rotating shifts that included night working (in a 3×8 hour pattern, containing a 20:00–4:00 night shift, a 4:00–12:00 morning shift and a 12:00–20:00 afternoon shift).^14^ Those who worked such a shift pattern were classified as working rotating shifts that involve night work. Information from 1990 was used in a minority of cases where values from 1989 were not available.

##### Workplace injuries

Information regarding workplace injuries was obtained from the company's medical examination files for sickness absence for the period 1978–2009; therefore, this is a measure of cumulative exposure to physical hazards. Whether individuals took at least 1 day off due to a workplace injury was medically certified by a company doctor. Sickness absence which was related to work tasks was classified as a workplace injury, whether this was an injury at work (‘accident de travail’) or a subsequent period off work as a result of the original injury (‘rechute’). The total number of episodes of absences for each participant due to injuries at work was calculated for the whole period. The variable is zero-inflated, so it was trichotomised into no injury episodes recorded, one injury episode and two or more injury episodes.

##### Chemical exposures

Exposures to chemicals were calculated for each participant using information from the company job-exposure matrix, which estimates, since the 1950s, each employee's estimated annual exposure to each of around 30 chemicals classified as known or suspected to cause cancer by the International Agency for Research on Cancer according to their occupational histories.^15^ ^16^ Record keeping was discontinued in 1998 when participants were no longer working in high-exposure posts.^17^ Cumulative exposures to chemical carcinogens over the career were measured by adding the number of different chemicals to which individuals were exposed in each year and adding these annual totals together. The median value was 70 years of accumulated chemical exposures, which would, for example, correspond to 10 years of exposure to each of seven chemicals. Individuals were regrouped into three categories: no exposure, exposed at or below the median level of those exposed and exposed at more than the median level.

#### Confounding factors

Two measures of social position: occupational grade and social class in 1989 were obtained from company personnel records. The three occupational grades are high-level, mid-level and low-level employees, categories which correspond to limits on maximum salaries. Social class was measured using the first level of the French national classification of occupations, which is in four categories: (1) managers and professionals, (2) intermediate occupations, (3) low-level non-manual and (4) low-level manual.

### Statistical analysis

Multistate life table methods were used to estimate partial life expectancies and health expectancies. Partial life expectancy is an age constrained estimation of life expectancy, here constrained to ages 50–75 years, inclusive. The analyses were performed separately for each of the six physical occupational exposures. A first set of models estimated partial life expectancies only in relation to each physical occupational exposure and a second set controlled for social position, because previous work has demonstrated socioeconomic differences in health expectancy.^18^ Partial health expectancy between ages 50 and 75 was defined in two ways, as having good self-rated health and as having no chronic conditions. For self-rated health and chronic conditions, the dependent variable can take one of three values: (1) good health (or no chronic conditions), (2) poor health (or having one or more chronic conditions) or (3) death.

The Stochastic Population Analysis for Complex Events (SPACE) program in SAS V.9.4 was used to estimate multistate life table functions.^6^ Multinomial logistic models were used to estimate transition probabilities; transition tables with age-specific predicted probabilities of transition between health states are presented in online supplementary tables S1 and S2. For estimates of health expectancies using self-rated health, 4 transitions could occur from 1 year to the next: healthy to unhealthy, unhealthy to healthy, healthy to dead, unhealthy to dead. Death was an absorbing state, while the other states were reversible. For estimates of health expectancies using chronic health conditions, only 3 transitions were possible as, by definition, recovery from chronic conditions was not possible. It was assumed that the observed events were independent and that no missing events took place between successive observations. Using microsimulation, individual health trajectories for a simulated cohort of 100 000 people were generated from distributions of covariates at the starting point based on the observed prevalence by age, social position (social class and occupational grade) and level of the physical occupational exposure. Partial life expectancy, healthy life expectancy (life expectancy without chronic disease) and unhealthy life expectancy (life expectancy with chronic disease) from ages 50 to 75 were calculated as the average of these trajectories for each occupational exposure. Additionally, the proportion of time spent in good self-rated health or without chronic disease was calculated. In a final step, variance estimates were obtained from bootstrapping (500 replicates).

10.1136/oemed-2016-103804.supp1 supplementary tables

## Results

Participants' sociodemographic characteristics, levels of physical occupational exposures and health at the baseline year are displayed in table 1. While the frequency of the physical work exposures varied, some were common: Under 6% of participants reported regularly working a rotating shift pattern that involved night work, 18% of the sample had experienced at least one industrial injury necessitating one or more days of leave and 61% of the participants had been exposed to hazardous chemicals. At baseline, 81% of participants reported good self-rated health and 54% no chronic health conditions.

**Table 1:** Physical occupational exposures and baseline sociodemographic and health characteristics (GAZEL cohort, men, n=13 393)

Variable	n	Per cent
*Sociodemographics*		
Age group (years)		
50–54	12 651	94.5
55–59	506	3.8
60–64	172	1.3
65–69	52	0.4
70–74	12	0.1
Occupational grade (1989)		
Low	1736	13.0
Middle	7491	55.9
High	4166	31.1
Social class (1989)		
Managers and professionals	4022	30.0
Intermediate occupations	7378	55.1
Low-level non-manual	502	3.8
Low-level manual	1491	11.1
*Physical occupational exposures*		
Perceived ergonomic strain (1989/1990)		
No exposure	4611	34.4
Exposed, median or less	4886	36.5
Exposed, over median	3896	29.1
Perceived physical strain (1989/1990)		
Median or less	7715	57.6
Over median	5678	42.4
Perceived physical danger (1989/1990)		
No exposure	2829	21.1
Exposed, median or less	5447	40.7
Exposed, over median	5117	38.2
Rotating shifts involving night working (1989/1990)		
Never or occasionally	12 654	94.5
Regularly	739	5.5
Number of episodes of absence due to industrial injuries (1978–2009)		
None	11 047	82.5
One	1605	12.0
Two or more	741	5.5
Accumulated chemical exposures (1956–1998)		
No exposure	5205	38.9
Exposed, median or less	4123	30.8
Exposed, over median	4065	30.4
*Health*		
Self-rated health		
Good	10 792	80.6
Poor	2601	19.4
Chronic health conditions		
No	7227	54.0
Yes*	6166	46.0

*Presence of chronic health conditions includes illness reported at or before baseline.

Results from the multistate models before and after adjustment are displayed in table 2 for self-rated health and table 3 for chronic conditions. The results changed little after adjustment for social class and job grade; therefore, we describe only the adjusted results here. Participants reporting highest levels of ergonomic strain, more than the median level of perceived physical strain and one industrial injury had shorter partial life expectancies from ages 50 to 75 years by between 4 and 6 months than unexposed participants. No differences were observed for the other physical working conditions.

**Table 2:** Partial life expectancy, healthy and unhealthy life expectancies and proportion of life spent in good health between the ages of 50 and 75 in relation to physical working conditions (GAZEL cohort, men, n=13 393)

	Partial life expectancy between the ages of 50 and 75							
	Unadjusted				Adjusted for social class and job grade			
	Life expectancy (95% CI)	Healthy life expectancy (95% CI)	Unhealthy life expectancy (95% CI)	Proportion (%) spent in good health (95% CI)	Life expectancy (95% CI)	Healthy life expectancy (95% CI)	Unhealthy life expectancy (95% CI)	Proportion (%) spent in good health (95% CI)
Perceived ergonomic strain (1989/1990)								
No exposure	24.5 (24.3 to 24.6)	20.9 (20.7 to 21.2)	3.5 (3.3 to 3.7)	85.6 (85.0 to 86.4)	24.4 (24.3 to 24.6)	20.9 (20.7 to 21.2)	3.5 (3.3 to 3.7)	85.5 (84.8 to 86.4)
Exposed, median or less	24.5 (24.3 to 24.6)	20.4 (20.1 to 20.5)	4.1 (3.9 to 4.3)	83.2 (82.4 to 83.9)	24.5 (24.3 to 24.6)	20.4 (20.1 to 20.6)	4.1 (3.9 to 4.3)	83.1 (82.5 to 84.0)
Exposed, over median	24.1 (23.9 to 24.3)	19.4 (19.1 to 19.6)	4.7 (4.5 to 4.9)	80.4 (79.5 to 81.3)	24.1 (23.9 to 24.3)	19.4 (19.1 to 19.6)	4.7 (4.5 to 5.0)	80.4 (79.4 to 81.3)
Perceived physical strain (1989/1990)								
Median or less	24.5 (24.4 to 24.6)	21.0 (20.8 to 21.1)	3.5 (3.4 to 3.7)	85.6 (85.0 to 86.1)	24.5 (24.4 to 24.6)	20.9 (20.8 to 21.1)	3.6 (3.4 to 3.7)	85.5 (85.0 to 86.0)
Over median	24.1 (24.0 to 24.3)	19.3 (19.1 to 19.5)	4.9 (4.6 to 5.0)	79.9 (79.1 to 80.8)	24.2 (24.0 to 24.3)	19.3 (19.1 to 19.5)	4.9 (4.7 to 5.0)	79.8 (79.1 to 80.7)
Perceived physical danger (1989/1990)								
No exposure	24.3 (24.1 to 24.5)	20.4 (20.2 to 20.8)	3.8 (3.6 to 4.0)	84.2 (83.4 to 85.2)	24.3 (24.0 to 24.5)	20.4 (20.1 to 20.7)	3.9 (3.6 to 4.0)	84.1 (83.4 to 85.0)
Exposed, median or less	24.4 (24.3 to 24.6)	20.5 (20.3 to 20.7)	3.9 (3.7 to 4.1)	84.0 (83.4 to 84.8)	24.4 (24.3 to 24.5)	20.5 (20.3 to 20.7)	3.9 (3.7 to 4.1)	83.9 (83.4 to 84.7)
Exposed, over median	24.4 (24.2 to 24.5)	19.9 (19.7 to 20.1)	4.4 (4.2 to 4.6)	81.8 (81.1 to 82.6)	24.3 (24.2 to 24.5)	19.9 (19.6 to 20.1)	4.4 (4.3 to 4.6)	81.8 (80.9 to 82.5)
Rotating shifts involving night working (1989/1990)								
Never or occasionally	24.4 (24.3 to 24.5)	20.3 (20.1 to 20.4)	4.1 (4.0 to 4.2)	83.2 (82.6 to 83.6)	24.4 (24.3 to 24.5)	20.3 (20.1 to 20.4)	4.1 (4.0 to 4.3)	83.1 (82.6 to 83.6)
Regularly	24.1 (23.5 to 24.4)	20.7 (19.9 to 21.1)	3.4 (3.1 to 3.9)	85.8 (83.6 to 87.1)	24.1 (23.6 to 24.5)	20.6 (19.9 to 21.1)	3.5 (3.1 to 4.0)	85.5 (83.6 to 87.3)
Number of episodes of absence due to industrial injuries (1978–2009)								
None	24.4 (24.3 to 24.5)	20.6 (20.4 to 20.8)	3.9 (3.7 to 4.0)	84.3 (83.7 to 84.8)	24.5 (24.3 to 24.5)	20.6 (20.4 to 20.7)	3.9 (3.7 to 4.0)	84.2 (83.8 to 84.7)
One	23.9 (23.6 to 24.1)	19.0 (18.6 to 19.4)	4.9 (4.5 to 5.2)	79.7 (78.3 to 81.0)	23.8 (23.6 to 24.2)	19.1 (18.6 to 19.5)	4.8 (4.5 to 5.2)	79.9 (78.4 to 81.1)
Two or more	24.1 (23.6 to 24.6)	17.8 (17.2 to 18.8)	6.2 (5.4 to 6.7)	74.1 (72.2 to 77.5)	24.2 (23.6 to 24.5)	18.0 (17.3 to 18.6)	6.2 (5.7 to 6.7)	74.4 (72.2 to 76.6)
Accumulated chemical exposures (1956–1998)								
No exposure	24.4 (24.3 to 24.6)	20.5 (20.3 to 20.7)	3.9 (3.8 to 4.1)	83.9 (83.1 to 84.6)	24.4 (24.3 to 24.6)	20.5 (20.3 to 20.7)	3.9 (3.8 to 4.1)	84.0 (83.2 to 84.6)
Exposed, median or less	24.4 (24.3 to 24.6)	20.5 (20.3 to 20.7)	3.9 (3.8 to 4.1)	83.9 (83.3 to 84.6)	24.4 (24.2 to 24.6)	20.4 (20.2 to 20.7)	3.9 (3.7 to 4.2)	83.8 (82.9 to 84.7)
Exposed, over median	24.2 (24.1 to 24.4)	19.8 (19.5 to 20.0)	4.4 (4.2 to 4.6)	81.7 (80.9 to 82.6)	24.2 (24.0 to 24.4)	19.7 (19.5 to 20.0)	4.5 (4.2 to 4.7)	81.6 (80.7 to 82.5)

**Table 3:** Partial life expectancy, life expectancies with and without chronic disease and proportion of life spent without chronic disease between the ages of 50 and 75 in relation to physical working conditions (GAZEL cohort, men, n=13 393)

	Partial life expectancy between the ages of 50 and 75							
	Unadjusted				Adjusted for social class and job grade			
	Life expectancy (95% CI)	Life expectancy without chronic disease (95% CI)	Life expectancy with chronic disease (95% CI)	Proportion (%) spent without chronic disease (95% CI)	Life expectancy (95% CI)	Life expectancy without chronic disease (95% CI)	Life expectancy with chronic disease (95% CI)	Proportion (%) spent without chronic disease (95% CI)
Perceived ergonomic strain (1989/90)								
No exposure	24.5 (24.3 to 24.6)	8.0 (7.7 to 8.3)	16.5 (16.2 to 16.8)	32.6 (31.5 to 34.0)	24.5 (24.3 to 24.6)	8.0 (7.7 to 8.3)	16.5 (16.2 to 16.8)	32.6 (31.5 to 33.7)
Exposed, median or less	24.5 (24.3 to 24.6)	7.1 (6.9 to 7.5)	17.4 (17.0 to 17.6)	29.0 (28.3 to 30.7)	24.5 (24.3 to 24.6)	7.2 (6.9 to 7.5)	17.3 (16.9 to 17.6)	29.4 (28.3 to 30.7)
Exposed, over median	24.1 (23.9 to 24.3)	5.8 (5.5 to 6.2)	18.3 (17.9 to 18.6)	24.0 (23.0 to 25.7)	24.1 (23.9 to 24.3)	5.9 (5.5 to 6.1)	18.3 (17.9 to 18.6)	24.2 (23.0 to 25.5)
Perceived physical strain (1989/1990)								
Median or less	24.6 (24.4 to 24.6)	7.7 (7.4 to 7.9)	16.9 (16.6 to 17.1)	31.2 (30.3 to 32.2)	24.5 (24.4 to 24.7)	7.7 (7.4 to 7.9)	16.8 (16.6 to 17.1)	31.4 (30.3 to 32.2)
Over median	24.2 (24.0 to 24.3)	6.2 (6.0 to 6.5)	17.9 (17.6 to 18.2)	25.8 (25.0 to 27.1)	24.1 (24.0 to 24.3)	6.3 (6.0 to 6.5)	17.8 (17.6 to 18.1)	26.1 (24.9 to 27.1)
Perceived physical danger (1989/1990)								
No exposure	24.3 (24.1 to 24.5)	7.7 (7.4 to 8.2)	16.6 (16.1 to 17.0)	31.7 (30.5 to 33.8)	24.2 (24.0 to 24.5)	7.9 (7.5 to 8.1)	16.4 (16.1 to 16.8)	32.4 (30.8 to 33.6)
Exposed, median or less	24.4 (24.3 to 24.6)	7.5 (7.2 to 7.7)	16.9 (16.7 to 17.3)	30.8 (29.4 to 31.6)	24.4 (24.3 to 24.6)	7.5 (7.1 to 7.8)	16.9 (16.7 to 17.3)	30.6 (29.3 to 31.6)
Exposed, over median	24.4 (24.2 to 24.5)	6.3 (6.0 to 6.6)	18.1 (17.8 to 18.4)	25.8 (24.8 to 26.9)	24.3 (24.2 to 24.5)	6.3 (6.0 to 6.6)	18.0 (17.7 to 18.4)	25.9 (24.5 to 27.2)
Rotating shifts involving night working (1989/1990)								
Never or occasionally	24.4 (24.3 to 24.5)	7.0 (6.9 to 7.2)	17.3 (17.2 to 17.6)	28.8 (28.1 to 29.5)	24.4 (24.3 to 24.5)	7.0 (6.9 to 7.2)	17.3 (17.2 to 17.6)	28.9 (28.1 to 29.7)
Regularly	24.0 (23.6 to 24.5)	8.0 (6.9 to 8.8)	16.0 (15.2 to 17.1)	33.5 (29.2 to 36.5)	23.9 (23.6 to 24.5)	8.1 (7.1 to 8.8)	15.8 (15.2 to 17.0)	33.9 (29.6 to 36.5)
Number of episodes of absence due to industrial injuries (1978–2009)								
None	24.5 (24.4 to 24.6)	7.5 (7.3 to 7.7)	17.0 (16.7 to 17.1)	30.6 (29.9 to 31.5)	24.5 (24.4 to 24.5)	7.5 (7.3 to 7.7)	17.0 (16.7 to 17.2)	30.7 (29.9 to 31.5)
One	23.9 (23.7 to 24.1)	5.3 (4.8 to 5.4)	18.6 (18.0 to 19.0)	22.3 (20.3 to 24.5)	23.9 (23.6 to 24.2)	5.4 (4.9 to 5.9)	18.5 (18.1 to 19.0)	22.7 (20.6 to 24.5)
Two or more	24.1 (23.6 to 24.6)	4.1 (3.5 to 4.7)	20.0 (19.3 to 20.8)	16.9 (14.4 to 19.5)	24.1 (23.7 to 24.5)	3.9 (3.4 to 4.8)	20.2 (19.3 to 20.8)	16.3 (14.2 to 20.0)
Accumulated chemical exposures (1956–1998)								
No exposure	24.4 (24.3 to 24.6)	7.7 (7.5 to 8.0)	16.7 (16.5 to 17.0)	31.6 (30.5 to 32.8)	24.5 (24.3 to 24.6)	7.7 (7.4 to 8.0)	16.8 (16.4 to 17.0)	31.4 (30.3 to 32.6)
Exposed, median or less	24.4 (24.3 to 24.6)	7.3 (7.0 to 7.5)	17.1 (16.9 to 17.5)	29.9 (28.5 to 30.8)	24.4 (24.2 to 24.6)	7.3 (6.9 to 7.6)	17.1 (16.8 to 17.5)	29.8 (28.4 to 31.0)
Exposed, over median	24.2 (24.1 to 24.4)	6.1 (5.8 to 6.4)	18.1 (17.8 to 18.4)	25.2 (24.0 to 26.5)	24.2 (24.0 to 24.4)	6.1 (5.8 to 6.4)	18.1 (17.8 to 18.5)	25.2 (24.0 to 26.4)

In the case of rotating shifts involving night working, the number of years participants were expected to live in poor health (or with chronic illness) was fewer among those who worked rotating shifts regularly. The proportion of life spent in good self-rated health was higher for participants who reported regularly rotating shifts that involved night working; although the 95% CIs slightly overlapped, there appeared to be a relationship in the same direction for the chronic disease outcome (never or occasional rotating shifts: 28.9%, 95% CIs 28.1% to 29.7%, regular rotating shifts: 33.9%, 95% CI 29.6% to 36.5%).

**Figure 1:**
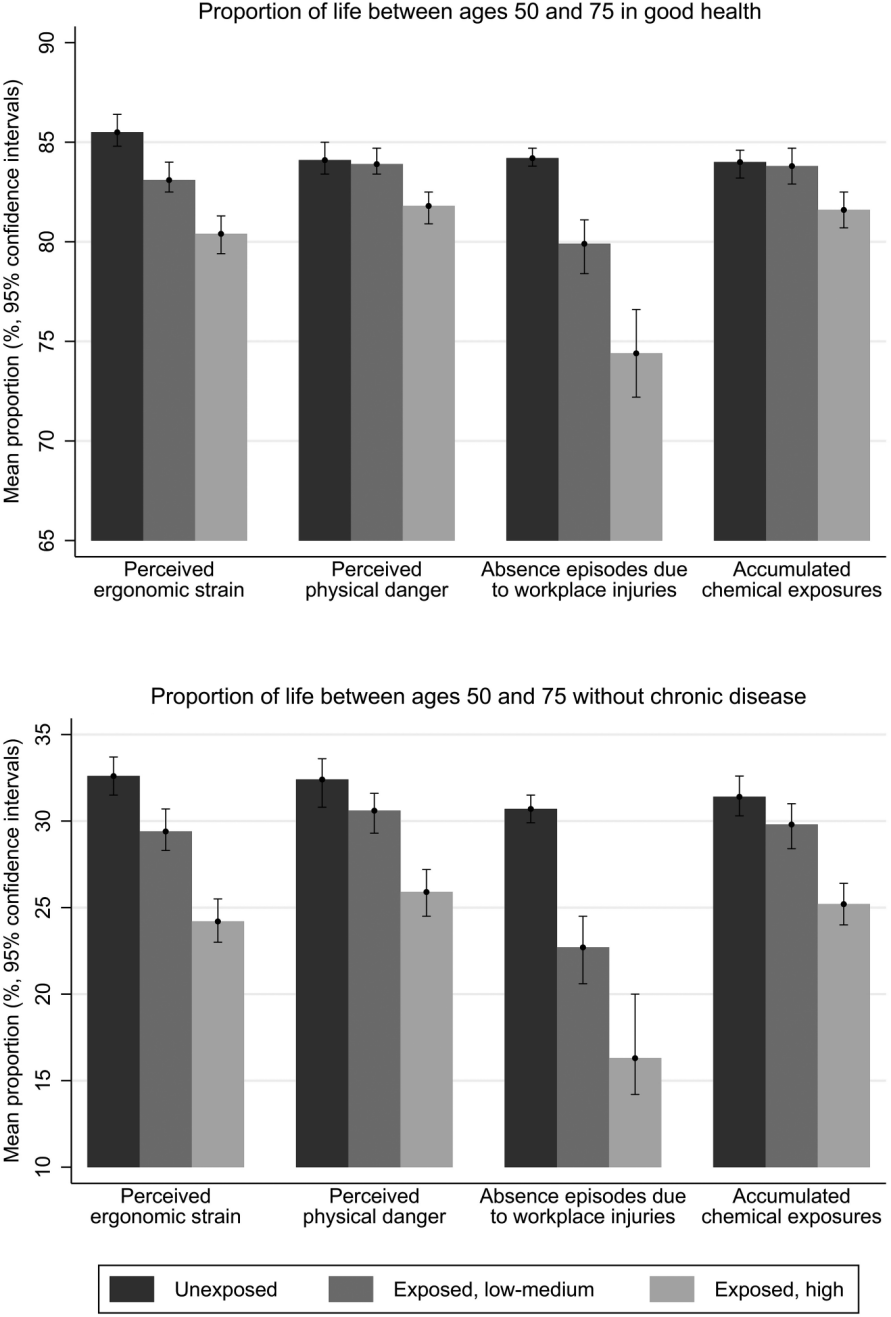
Proportion of life spent in good health or without chronic disease between the ages of 50 and 75 in relation to selected physical working conditions (GAZEL cohort, men, n=13 393). The categories for perceived ergonomic strain, perceived physical danger and accumulated chemical exposures are unexposed; exposed, median or less; exposed, over median. For absence episodes due to workplace injuries, the categories are no episodes of absence following workplace injury (unexposed), one episode of absence following workplace injury (exposed, low-medium) and two or more such episodes of absence (exposed, high).

Health expectancies as well as proportion of years spent in good health were generally associated in a graded manner with perceived ergonomic strain, perceived physical danger, industrial injuries and accumulated chemical exposures (table 2 and figure 1). Such gradients were observed more consistently in relation to the chronic illness outcome (table 3 and figure 1). Differences were particularly marked in the case of industrial injuries, where participants without a record of an industrial injury could expect to spend 30.7% of their life between 50 and 75 years without chronic disease, while the corresponding figure for those who had records of at least two episodes of absence as a result of industrial injuries was 16.3% (cf. figure 1 for self-rated health). Compared to those with less perceived physical strain, participants reporting greater perceived physical strain had shorter health expectancies (by 1.6 years in good self-rated health and 1.4 years without chronic disease) as well as a lower proportion of years in good self-rated health and without chronic disease.

## Discussion

This study used the multistate life table method with a prospective cohort and two measures of health expectancy: self-rated health and absence of chronic illness to estimate differences in partial healthy life expectancy in relation to a range of physical working conditions. People exposed to more strenuous and dangerous work could expect to spend fewer years in good health between the ages of 50 and 75. Additionally, there was some evidence of a double burden of shorter partial life expectancy and lower proportion of years lived in good health or without chronic illness for participants exposed to strenuous working conditions and industrial injuries.

Although, to the best of our knowledge, there is no previous work which analyses partial healthy life expectancy in relation to physical occupational exposures, the results obtained here are in line with research suggesting the health impacts of ergonomic strain and dangerous work. Exposures to toxic substances have been linked to chronic illnesses as well as declarations of poor health,^3^ ^5^ ^19^ while biomechanical hazards have been shown to cause chronic illness^4^ ^20^ ^21^ and also been associated with limitations to daily activities^22^ and raised hospital admission rates at older ages.^23^

In the case of regular shift work involving night working, a reverse trend was found such that employees who regularly worked rotating shifts had a higher proportion of years lived in good health compared to those who did not work rotating shifts. This result may have been affected by health selection, in which less healthy workers avoid rotating shift work involving night working or have moved out of such jobs into daytime roles.^24^

This study has some strengths. It uses two measures of health expectancy: self-rated health and chronic illnesses, which is valuable in a field characterised by heterogeneity in outcome measures.^18^ It uses prospective data with lengthy follow-up as a basis for estimating health expectancy and administrative data on working conditions as well as self-reports. The multistate life table method uses health and mortality data from the same survey which has the advantage of producing consistent transition probabilities between health states. However, the study also has several limitations. Four of the physical occupational exposures were self-reports from 1989 or 1990; there may be misclassification of exposures if employees' working conditions had changed since this time. Members of the GAZEL cohort, securely employed in a large organisation, are likely to be healthier and have safer working conditions than the population they represent, and there were health differences in attrition rates.^25^ Selection into certain occupations is likely to have been taking place, which may reduce the size of the effects observed or even reverse them, if healthier workers selected more strenuous and hazardous occupations or regular night-time working.^24^ In addition, although we controlled for two measures of social position, there is a possibility that other aspects of individuals' socioeconomic situation might confound the analyses. Extending the analysis to women could be potential future work, since some women in the GAZEL cohort were exposed to work injuries and ergonomic strain.^26^ Finally, health expectancy estimates assume stationary transition rates over time, like other life table-based measures. Therefore, these results do not apply to any specific cohort should age-specific rates be changing over time.

The relationship between physical working conditions and healthy life expectancy has particular relevance in the current context in which workers continue to report high levels of physical occupational exposures: nearly half of the participants in a recent European working conditions survey reported having to hold tiring or painful positions at work, and over one-third of workers reported handling heavy loads.^27^ In addition, since people in poor health tend to retire at younger ages,^28^ ^29^ this result has implications for governments seeking to extend working lives.

## Conclusions

There were differences by physical working conditions for partial healthy life expectancy between 50 and 75 years such that people exposed to more strenuous and dangerous work could generally expect to spend fewer years in good health. This result has implications for governments seeking to improve the health of older adults, since physical working conditions are modifiable factors.
