# Supplementary material for: Physical occupational exposures and health expectancies in a French occupational cohort
Source: Occup Environ Med. 2016 Sep 21;74(3):176–83. doi: 10.1136/oemed-2016-103804 (PMC5520272; doi:10.1136/oemed-2016-103804)
Supplement: supplementary tables [file oemed-2016-103804supp001.pdf]

Physical occupational exposures and health expectancies in a French occupational cohort

Supplementary Tables

Table S1: Odds ratios for self-reported health transitions from multinomial logistic models (GAZEL cohort, men, n=13393)

	Healthy to unhealthy	Unhealthy to healthy	Healthy to death	Unhealthy to death
	OR (95% CI)*	OR (95% CI)*	OR (95% CI)*	OR (95% CI)*
Perceived ergonomic strain (1989/90)				
No exposure	1.00	1.00	1.00	1.00
Exposed, median or less	1.13 (1.09; 1.18)	.94 (.89; .99)	.94 (.77; 1.14)	.87 (.71; 1.05)
Exposed, over median	1.23 (1.18; 1.29)	.95 (.90; 1.01)	1.06 (.86; 1.31)	.87 (.71; 1.08)
Perceived physical strain (1989/90)				
Median or less	1.00	1.00	1.00	1.00
Over median	1.25 (1.20; 1.29)	.83 (.80; .87)	1.20 (1.01; 1.42)	.86 (.73; 1.01)
Perceived physical danger (1989/90)				
No exposure	1.00	1.00	1.00	1.00
Exposed, median or less	1.09 (1.04; 1.15)	1.14 (1.07; 1.21)	.89 (.72; 1.10)	.94 (.76; 1.16)
Exposed, over median	1.13 (1.08; 1.19)	1.12 (1.06; 1.20)	.76 (.60; .95)	.85 (.67; 1.07)
Rotating shifts involving night working (1989/90)				
Never or occasionally	1.00	1.00	1.00	1.00
Regularly	.87 (.80; .94)	1.00 (.90; 1.11)	1.37 (1.00; 1.89)	1.29 (.91; 1.85)
Number of episodes of absence due to industrial injuries (1978–2009)				
None	1.00	1.00	1.00	1.00
One	1.16 (1.10; 1.22)	.89 (.83; .95)	1.43 (1.14; 1.78)	.91 (.72; 1.15)
Two or more	1.32 (1.22; 1.42)	.74 (.67; .80)	1.03 (.71; 1.50)	.69 (.50; .97)
Accumulated chemical exposures (1956–1998)				
No exposure	1.00	1.00	1.00	1.00
Exposed, median or less	.94 (.90; .99)	1.05 (.99; 1.11)	1.10 (.90; 1.35)	.83 (.68; 1.01)
Exposed, over median	.99 (.95; 1.04)	1.11 (1.05; 1.18)	1.03 (.82; 1.29)	.81 (.65; 1.01)

*Adjusted for social class and job grade.

Table S2: Odds ratios for chronic condition transitions from multinomial logistic models (GAZEL cohort, men, n=13393)

	Healthy to unhealthy<br>OR (95% CI)*	Healthy to death<br>OR (95% CI)*	Unhealthy to death<br>OR (95% CI)*
Perceived ergonomic strain (1989/90)			
No exposure	1.00	1.00	1.00
Exposed, median or less	1.01 (.95; 1.09)	.94 (.71; 1.24)	.95 (.81; 1.11)
Exposed, over median	1.03 (.95; 1.13)	1.22 (.90; 1.67)	.97 (.82; 1.15)
Perceived physical strain (1989/90)			
Median or less	1.00	1.00	1.00
Over median	1.05 (.98; 1.12)	1.26 (.99; 1.60)	1.10 (.97; 1.26)
Perceived physical danger (1989/90)			
No exposure	1.00	1.00	1.00
Exposed, median or less	1.03 (.95; 1.12)	.89 (.66; 1.20)	.89 (.74; 1.05)
Exposed, over median	1.05 (.96; 1.15)	.79 (.57; 1.11)	.78 (.65; .94)
Rotating shifts involving night working (1989/90)			
Never or occasionally	1.00	1.00	1.00
Regularly	.87 (.76; 1.00)	1.16 (.73; 1.85)	1.32 (1.00; 1.73)
Number of episodes of absence due to industrial injuries (1978–2009)			
None	1.00	1.00	1.00
One	1.13 (1.02; 1.26)	1.63 (1.15; 2.29)	1.12 (.93; 1.34)
Two or more	1.34 (1.14; 1.58)	1.15 (.60; 2.20)	.94 (.71; 1.23)
Accumulated chemical exposures (1956–1998)			
No exposure	1.00	1.00	1.00
Exposed, median or less	.99 (.92; 1.06)	.98 (.74; 1.29)	.90 (.77; 1.06)
Exposed, over median	1.06 (.98; 1.16)	.75 (.54; 1.06)	.90 (.75; 1.07)

*Adjusted for social class and job grade.
